# Interpretable Machine Learning Model for Predicting 30‐Day Readmission in Advanced Heart Failure Patients: Synergistic Assessment of Inflammatory and Metabolic Biomarkers

**DOI:** 10.1155/cdr/2307901

**Published:** 2026-03-08

**Authors:** Baohe Zang, Chengyu Li, Min Zhou, Yali Chao

**Affiliations:** ^1^ Department of Intensive Care Medicine, The Affiliated Hospital of Xuzhou Medical University, Xuzhou, Jiangsu, China, xzmc.edu.cn; ^2^ Department of Orthopedics, The Affiliated Hospital of Xuzhou Medical University, Xuzhou, Jiangsu, China, xzmc.edu.cn

**Keywords:** 30-day readmission, advanced heart failure, CRP, machine learning, TyG-BMI

## Abstract

**Background:**

Patients with advanced heart failure (AdHF) face a high risk of early readmission, leading to poor outcomes and increased healthcare burden. Early identification of high‐risk individuals remains a clinical challenge.

**Methods:**

This retrospective study included 769 AdHF patients from the Affiliated Hospital of Xuzhou Medical University, with an independent external validation performed on a cohort of 495 AdHF patients from Shanghai Tenth People′s Hospital. After handling missing data via multiple imputation, the dataset was randomly split into training and validation sets in a 7:3 ratio. Class imbalance was addressed by applying the synthetic minority oversampling technique (SMOTE) exclusively within the training set, followed by the least absolute shrinkage and selection operator (LASSO)‐based feature selection. Seven machine learning models were developed. Model performance was evaluated using the area under the receiver operating characteristic curve (AUC), calibration curve, accuracy, sensitivity, specificity, F1 score, and Brier score, among others. SHAP values and a web‐based dynamic nomogram were used for model interpretation and clinical application.

**Results:**

The 30‐day readmission rate was 30.9%. RF achieved the highest AUC (0.85), accuracy (0.79), and the lowest Brier score (0.159). Key predictors included C‐reactive protein (CRP), triglyceride‐glucose‐body mass index (TYG‐BMI), neutrophil‐to‐lymphocyte ratio (NLR), age, New York Heart Association (NYHA) class, atrial fibrillation (AF), comorbidity count, and angiotensin‐converting enzyme inhibitors/angiotensin receptor blockers/angiotensin receptor–neprilysin inhibitors (ACEI/ARB/ARNI) use. SHAP analysis confirmed the importance of inflammatory and metabolic markers. A web‐based nomogram was constructed to allow interactive risk prediction.

**Conclusion:**

This study presents a robust and interpretable model for predicting a 30‐day readmission in AdHF patients, highlighting the role of inflammation, metabolism, and comorbidity burden. The model can assist clinicians in risk stratification and personalized postdischarge management.

## 1. Introduction

Advanced Heart Failure (AdHF) represents the terminal phenotype of cardiovascular diseases, characterized by severely impaired cardiac function, progressive myocardial remodeling, and multiorgan dysfunction [1, 2]. Epidemiological data indicate that AdHF accounts for approximately 1%–10% of all heart failure (HF) cases, with a steadily increasing prevalence [3]. Although the application of ventricular assist devices (VADs) and heart transplantation has improved survival in selected patients in recent years, AdHF remains associated with high readmission rates, substantially elevated healthcare costs, and poor clinical outcomes. Readmission events not only signify cardiac decompensation but are also closely associated with various comorbidities, such as acute kidney injury, infections, and excessive activation of the neurohormonal system [4, 5]. It is noteworthy that the risk of readmission in patients with AdHF exhibits substantial heterogeneity, and accurate prediction requires comprehensive consideration of sociodemographic factors, comorbidities, inflammatory and metabolic biomarkers, and prescribed medications. Therefore, developing precision prediction tools based on multidimensional indicators has become a critical challenge for optimizing risk stratification and management in AdHF.

It is well established that inflammatory responses and metabolic dysregulation play a pivotal role in the progression of HF [6, 7]. Among various inflammatory biomarkers, C‐reactive protein (CRP), an acute‐phase reactant, has been independently associated with accelerated ventricular remodeling and an increased risk of readmission [8, 9]. In recent years, novel inflammatory indices such as the neutrophil‐to‐lymphocyte ratio (NLR) and the platelet‐to‐lymphocyte ratio (PLR) have garnered growing attention for their ability to reflect systemic inflammation and immune imbalance. A study has shown that elevated NLR levels are significantly associated with increased inflammatory markers in patients with HF, and are linked to worse outcomes, including higher all‐cause mortality and HF‐related hospitalizations [10]. In terms of metabolic dysregulation, the triglyceride‐glucose (TyG) index and its derivative, triglyceride‐glucose–body mass index (TYG‐BMI), have been validated as effective markers for assessing insulin resistance (IR) and lipotoxic myocardial injury [11, 12]. A study by Lyu et al. identified TyG‐BMI as a significant prognostic indicator in HF patients with coronary heart disease (CHD), showing a nonlinear association with all‐cause mortality and HF readmission [13]. However, most studies to date have analyzed these biomarkers in isolation or employed simple statistical models, with limited research conducted specifically in populations with AdHF. This approach overlooks the complex interactions and nonlinear associations among these factors.

Interpretable machine learning (ML) offers a transformative approach to uncovering the relationships among these biomarkers. Unlike traditional regression models, certain ML algorithms, particularly tree‐based ensembles such as random forest (RF) and eXtreme Gradient Boosting (XGBoost), can be effectively interpreted using SHapley Additive Explanations (SHAP). SHAP provides a theoretically grounded framework to quantify the contribution of each predictor to individual predictions. In this study, we innovatively integrated inflammatory and metabolic biomarkers into an interpretable ML framework to predict 30‐day readmission in patients with AdHF. By elucidating the synergistic effects of these biomarkers and developing an online predictive model, this work is aimed at advancing the implementation of personalized precision medicine strategies.

## 2. Methods

### 2.1. Study Design and Data Source

This retrospective cohort study utilized electronic medical records from the Affiliated Hospital of Xuzhou Medical University. In addition, an independent external validation cohort was obtained from Shanghai Tenth People′s Hospital to assess the generalizability and robustness of the model. Patient data were retrospectively retrieved from January 2015 to December 2024, encompassing admissions to the intensive care unit (ICU) and cardiology department. Follow‐up extended for 30 days postdischarge. To safeguard patient confidentiality, all data underwent anonymization and strict adherence to data protection regulations. The study protocol complied with the Declaration of Helsinki and received ethical approval from the institutional review boards of both the Affiliated Hospital of Xuzhou Medical University (XYFY2025‐KL089‐01) and Shanghai Tenth People′s Hospital (SHSY‐LYZX‐220). Given its retrospective nature and reliance on pre‐existing clinical records, the IRB waived the requirement for informed consent.

Inclusion criteria were (1) patients fulfilling the 2018 position statement on AdHF by the Heart Failure Association of the European Society of Cardiology (ESC) diagnostic criteria for AdHF and (2) age of ≥ 18 years. Exclusion criteria comprised (1) loss to follow‐up within 30 days; (2) > 30% missing values for key variables; (3) terminal malignancy with < 30 days; and (4) prior heart transplantation, as these patients no longer represent the natural disease course of AdHF and have distinct readmission etiologies.

### 2.2. Variable Definitions

#### 2.2.1. Outcome

Patients enrolled in the study were followed for 30 days after discharge through medical record review, outpatient visits, and telephone contact. The follow‐up period ended on January 30, 2025. The primary outcome was an unplanned all‐cause readmission within 30 days after discharge, defined as any unanticipated hospitalization due to worsening clinical condition, excluding planned or elective admissions for scheduled procedures (e.g., device implantation, rehabilitation, or routine follow‐up), or scheduled intravenous pharmacological therapies (e.g., levosimendan infusion). Death events occurring within 30 days were recorded separately but were not counted as readmissions to ensure outcome specificity. The 30‐day all‐cause readmission outcome includes unplanned hospitalizations at any healthcare facility, not limited to our institution. Information on readmissions to other hospitals was obtained through structured telephone interviews with patients or their caregivers within 30 days postdischarge.

#### 2.2.2. Predictors

A total of 47 variables potentially influencing the outcome were included in this study, covering sociodemographic characteristics, vital signs, therapy, echocardiographic findings, prescribed medications, laboratory results, comorbidities, and inflammatory and metabolic biomarkers. Inflammatory indices included NLR, PLR, monocyte‐to‐lymphocyte ratio (MLR), systemic immune‐inflammation index (SII), and systemic inflammation response index (SIRI). Metabolic markers included the TyG index and TyG‐BMI. Blood samples for all hematological and biochemical indicators were drawn within 24 h of hospital admission (typically on admission or early the following morning). Laboratory results derived from these samples were included in the analysis regardless of reporting delay. The number of comorbidities was defined as the count of pre‐existing chronic conditions documented in the discharge diagnosis, limited to the following eight clinically relevant conditions: hypertension, myocardial infarction (MI), atrial fibrillation (AF), stroke, diabetes mellitus (DM), chronic obstructive pulmonary disease (COPD), chronic kidney disease (CKD), and anemia.

The calculation formulas for these indices are as follows:

1. NLR = neutrophils/lymphocytes
2. PLR = platelets/lymphocytes
3. MLR = monocytes/lymphocytes
4. SII = (platelet∗neutrophil)/lymphocyte
5. SIRI = (neutrophil∗monocyte)/lymphocyte
6. TYG index = ln [(triglyceride (TG) level (mg/dL) × fasting blood glucose (FBG) level (mg/dL))]/2
7. TYG‐BMI = TYG index × weight (kg)/height (m)^2^

### 2.3. ML Model Development and Evaluation

The ML pipeline—including data splitting, oversampling, feature selection, model training, performance evaluation, and interpretability analysis—was implemented in Python (Version 3.9.13). Data preprocessing involved multiple imputation using the multivariate imputation by chained equations (MICE) method for missing values, followed by feature standardization. The dataset was randomly split into training and validation sets in a 7:3 ratio, with the former used for model development and the latter for performance evaluation. To address class imbalance, the synthetic minority oversampling technique (SMOTE) was applied strictly to the training set after data splitting, ensuring that the validation set remained untouched during model training. We acknowledge that SMOTE may alter the marginal distributions of predictors and potentially affect probability calibration. To mitigate this, isotonic calibration was explored as a post hoc adjustment. However, the final model interpretation (SHAP and nomogram) was based on the original RF to preserve clinical interpretability and feature contribution fidelity. Variable selection and dimensionality reduction were then performed using the least absolute shrinkage and selection operator (LASSO) with tenfold cross‐validation. LASSO feature selection was conducted exclusively within the training set to avoid data leakage from the validation data. Following feature selection via LASSO, we implemented seven ML algorithms: RF, decision tree (DT), XGBoost, support vector machine (SVM), logistic regression (LR), LightGBM, and multilayer perceptron (MLP).

The predictive performance of each model was assessed using a comprehensive set of metrics, including the area under the receiver operating characteristic curve (AUC), the area under the precision‐recall curve (AUPRC), calibration plots, decision curve analysis (DCA), overall accuracy, recall (sensitivity), specificity, positive predictive value (PPV), negative predictive value (NPV), F1 score, and Brier score.

### 2.4. Interpretability Analysis and Dynamic Nomogram Creation

For the interpretability analysis of the model, the SHAP method was employed for a global interpretation. By calculating the SHAP values of each feature, the contribution degree of each feature to the prediction result was quantified, revealing the potential associations between various types of indicators and the readmission risk.

The development of the dynamic nomogram was carried out using R (Version 3.6.4). Based on the results of model training and evaluation, a dynamic nomogram was further plotted, integrating the key predictive factors into a visual decision‐making tool. The nomogram quantifies the weights of each variable through a scoring system, enabling personalized prediction of the 30‐day readmission probability of patients and assisting in clinical risk stratification and decision‐making.

### 2.5. Statistical Analysis

All descriptive and univariate analyses in this section were performed using IBM SPSS Statistics (Version 23.0). Continuous variables were reported as mean ± standard deviation (X ± SD) for normally distributed data, or as median with interquartile range (P25, P75) for nonnormally distributed data. Categorical variables were summarized as counts and percentages. Between‐group comparisons were performed using the independent samples *t*‐test or Mann–Whitney *U* test for continuous variables, and the chi‐square test or Fisher′s exact test for categorical variables. A *p* value of < 0.05 was considered statistically significant.

## 3. Results

### 3.1. Study Population

**Figure 1:**
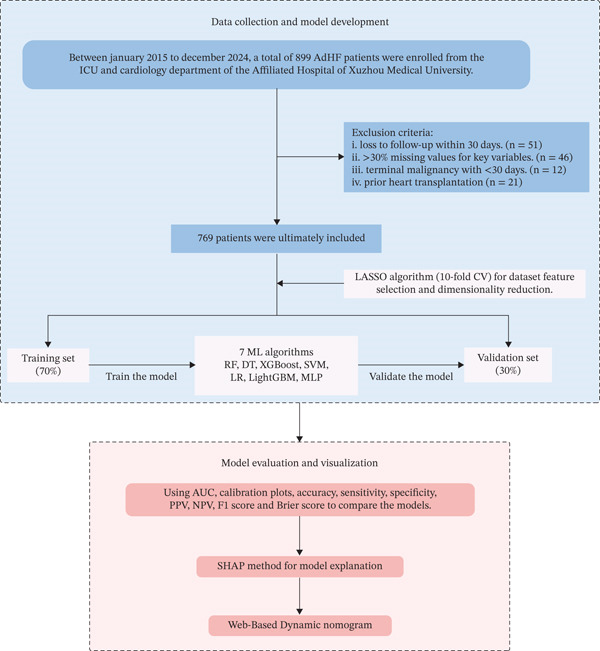
Flow chart of the study design. Abbreviations: AdHF, advanced heart failure; ICU, intensive care unit; LASSO, least absolute shrinkage and selection operator; CV, cross‐validation; ML, machine learning; RF, random forest; DT, decision tree; XGBoost, eXtreme Gradient Boosting; SVM, support vector machine; LR, logistic regression; LightGBM, light gradient boosting machine; MLP, multilayer perceptron; AUC, the area under the receiver‐operating characteristic; PPV, positive predictive value; NPV, negative predictive value; SHAP, SHapley Additive exPlanations.

A total of 899 patients were initially screened, and 769 were finally included after applying the exclusion criteria (Figure 1). Among them, 238 patients experienced 30‐day readmission, whereas 531 did not; detailed baseline characteristics are summarized in Table 1. Comparative analysis showed that the readmission group was older (72.93 ± 10.66 vs. 69.19 ± 12.23 years), had a slightly higher body mass index (BMI, 23.93 vs. 23.44 kg/m^2^), and a greater proportion of New York Heart Association (NYHA) Class IV patients (51.7% vs. 40.5%). AF was more prevalent in this group (35.7% vs. 25.8%), as was the presence of ≥ 2 comorbidities. Meanwhile, the use of angiotensin‐converting enzyme inhibitors/angiotensin receptor blockers/angiotensin receptor–neprilysin inhibitors (ACEI/ARB/ARNI) and beta‐blockers was lower. All aforementioned variables demonstrated significant differences (*p* < 0.05).

**Table 1:** Baseline characteristics of non‐readmission and readmission groups.

Variables	Non‐readmission group (*n* = 531)	Readmission group (*n* = 238)	*p* − *value*
Age	69.19 ± 12.23	72.93 ± 10.66	< 0.001
Gender (*n*, %)			0.412
Male	291 (54.8%)	138 (58.0%)	
Female	240 (45.2%)	100 (42.0%)	
BMI, kg/m^2^	23.44 (20.79, 25.82)	23.93 (21.48, 26.37)	0.035
SBP (mmHg)	121 (106, 139)	124 (108, 1400)	0.113
DBP (mmHg)	74 (65, 84)	74 (66, 83)	0.901
HR	84 (71, 97)	83 (70, 98)	0.897
Smoking (*n*, %)	148 (27.9%)	73 (30.7%)	0.428
Drinking (*n*, %)	49 (9.2%)	19 (8.0%)	0.574
NYHA class (*n*, %)			0.004
III	316 (59.5%)	115 (48.3%)	
IV	215 (40.5%)	123 (51.7%)	
Past medical history (*n*, %)			
Hypertension	228 (42.9%)	118 (49.6%)	0.087
MI	84 (15.8%)	50 (21.0%)	0.079
AF	137 (25.8%)	85 (35.7%)	0.005
Stroke	48 (9.0%)	24 (10.1%)	0.646
DM	137 (25.8%)	73 (30.7%)	0.161
COPD	37 (7.0%)	17 (7.1%)	0.930
CKD	53 (10.0%)	32 (13.4%)	0.157
Anemia	54 (10.2%)	34 (14.3%)	0.097
Number of comorbidities			0.007
0	108 (20.3%)	27 (11.3%)	
1	201 (37.9%)	79 (33.2%)	
2	121 (22.8%)	69 (29.0%)	
3	73 (13.7%)	43 (18.1%)	
4	24 (4.5%)	16 (6.7%)	
5	4 (0.8%)	4 (1.7%)	
PCI	111 (20.9%)	53 (22.3%)	0.669
Pacemaker	43 (8.1%)	26 (10.9%)	0.205
LVEF (%)	42.00 (27.00, 54.00)	45.00 (28.00, 55.00)	0.619
LVEDd	64.00 (55.00, 74.00)	62.75 (55.00, 70.00)	0.205
Indices			
NLR	3.26 (2.26, 4.91)	4.09 (2.39, 6.41)	< 0.001
PLR	134.00 (98.04, 186.67)	147.84 (101.40, 240.42)	0.024
MLR	0.35 (0.26, 0.52)	0.44 (0.27, 0.68)	< 0.001
SII	604.71 (371.37, 941.47)	664.51 (409.29, 1289.24)	0.014
SIRI	1.59 (1.00, 2.59)	1.97 (1.09, 3.98)	0.001
TYG	8.62 (8.22, 9.08)	8.66 (8.23, 9.10)	0.495
TYG‐BMI	201.05 (176.89, 229.10)	206.05 (183.51, 235.94)	0.052
SCr (umol/L)	90.00 (72.00, 118.00)	97.00 (80.00, 131.50)	0.003
UA (umo/L)	394.00 (309.00, 481.00)	379.50 (316.75, 469.00)	0.446
TG (mmol/L)	1.27 (0.89, 1.79)	1.24 (0.91, 1.69)	0.761
TC (mmol/L)	3.69 (3.03, 4.43)	3.74 (2.98, 4.58)	0.742
LDL‐C (mmol/L)	1.96 (1.44, 2.72)	2.02 (1.38, 2.67)	0.835
FBG (mmol/L)	5.30 (4.67, 6.86)	5.54 (4.67, 7.33)	0.279
CRP (mg/L)	5.34 (1.56, 17.61)	17.03 (7.70, 33.15)	< 0.001
BNP (pg/mL)	1113 (507, 2463)	1263 (661, 2673)	0.029
Inotropic drugs (*n*, %)			
Diuretic	434 (81.7%)	193 (81.1%)	0.833
Aldosterone‐receptor blocker	385 (72.5%)	172 (72.3%)	0.946
Nitrate esters	80 (15.1%)	44 (18.5%)	0.233
Digitonin	133 (25.0%)	54 (22.7%)	0.481
ACEI/ARB/ARNI	384 (72.3%)	136 (57.1%)	< 0.001
Beta‐blockers	337 (63.5%)	130 (54.6%)	0.020
Antiplatelet drugs	230 (43.3%)	117 (49.2%)	0.132
Anticoagulant	171 (32.2%)	75 (31.5%)	0.849
Statin	296 (55.7%)	150 (63.0%)	0.059
CCB	39 (7.3%)	18 (7.6%)	0.915

Abbreviations: ACEI, angiotensin‐converting enzyme inhibitor; AF, atrial fibrillation; ARB, angiotensin receptor blockers; ARNI, angiotensin receptor–neprilysin inhibitor; BMI, body mass index; BNP, brain natriuretic peptide; CCB, calcium channel blocker; CKD, chronic kidney disease; COPD, chronic obstructive pulmonary disease; CRP, C‐reactive protein; DBP, diastolic blood pressure; DM, diabetes mellitus; FBG, fasting blood glucose; HR, heart rate; LDL‐C, low‐density lipoprotein cholesterol; LVEDd, left ventricular end‐diastolic dimension; LVEF, left ventricular ejection fraction; MI, myocardial infarction; MLR, monocyte‐to‐lymphocyte ratio; NLR, neutrophil‐to‐lymphocyte ratio; NYHA, New York Heart Association; PCI, percutaneous coronary intervention; PLR, platelet‐to‐lymphocyte ratio; SBP, systolic blood pressure; Scr: serum creatinine; SII, systemic immune‐inflammation index; SIRI, systemic inflammation response index; TC, total cholesterol; TG, triglyceride; TYG, triglyceride‐glucose; TYG‐BMI, triglyceride‐glucose body mass index.

Inflammatory markers—including the NLR, PLR, MLR, SII, SIRI, and CRP—were all significantly elevated in the readmission group. In addition, serum creatinine (Scr) and B‐type natriuretic peptide (BNP) levels were also higher (all *p* < 0.05). The TyG‐BMI was slightly higher in the readmission group (206.05 vs. 201.05, *p* = 0.052), approaching statistical significance and warranting further investigation within the modeling framework.

### 3.2. Feature Selection

**Figure 2:**
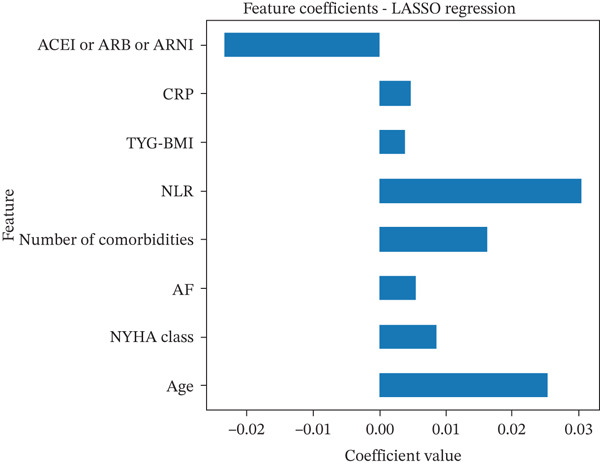
Using LASSO regression to select features with nonzero coefficients. Abbreviations: LASSO, least absolute shrinkage and selection operator; ACEI, angiotensin‐converting enzyme inhibitor; ARB, angiotensin receptor blockers; ARNI, angiotensin receptor–neprilysin inhibitor; CRP, C‐reactive protein; TYG‐BMI, triglyceride‐glucose–body mass index; NLR, the neutrophil‐to‐lymphocyte ratio; AF, atrial fibrillation; NYHA, New York Heart Association.

In this study, feature selection was performed using LASSO regression with tenfold cross‐validation (*C* *V* = 10), as shown in Figure 2. Age and the number of comorbidities exhibited relatively large coefficient values, indicating their prominent roles in predicting a 30‐day readmission risk. Among inflammatory and metabolic indicators, NLR was particularly influential, along with CRP and TyG‐BMI, all of which serve as important physiological markers that reflect the patient′s overall condition and contribute meaningfully to risk assessment. Additionally, the use of ACEI/ARB/ARNI, presence of AF, and NYHA classification were also retained as key predictors, jointly supporting accurate readmission prediction.

### 3.3. Performance of ML Models

As shown in Figure 3 and Table 2, the seven ML models exhibited marked differences in predicting 30‐day readmission. The RF model achieved the best overall performance with an AUC of 0.85 (95% CI: 0.81–0.89), significantly outperforming DT (*A* *U* *C* = 0.68) and LR (*A* *U* *C* = 0.67). The full set of hyperparameters for the final RF model is provided in Table S1. Specifically, RF achieved an accuracy of 0.79, a sensitivity of 0.79, a specificity of 0.78, and the lowest Brier score (0.159), indicating a minimal prediction error. Calibration analysis revealed that the predicted probabilities of RF closely matched the observed outcomes, aligning well with the ideal calibration curve. Quantitative calibration metrics confirmed excellent agreement, with an intercept of −0.0264 and a slope of 1.0312. The AF model achieved the highest AUPRC (0.696), indicating superior ability to identify high‐risk patients compared to other models (Table 2).

Figure 3ROC and calibration curves of seven machine learning models. (a) ROC curve. (b) Calibration curve. Abbreviations: AUC, the area under the receiver‐operating characteristic; CI, confidence interval; RF, random forest; DT, decision tree; XGBoost, eXtreme Gradient Boosting, SVM, support vector machine; LR, logistic regression; LightGBM, light gradient boosting machine; MLP, multilayer perceptron.

**Figure:**
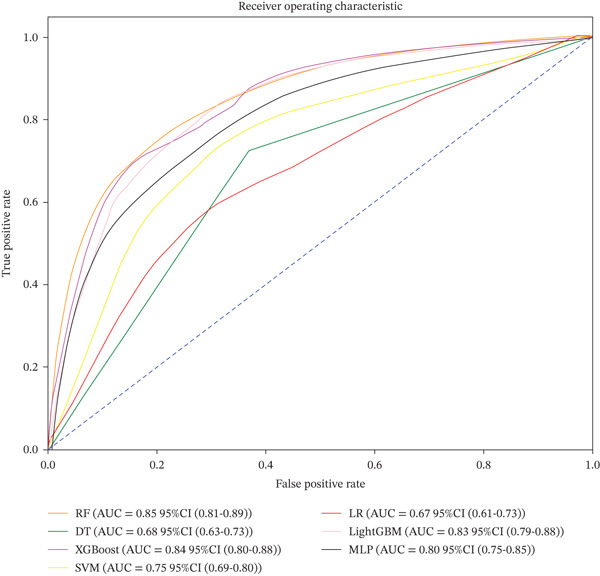
(a)

**Figure:**
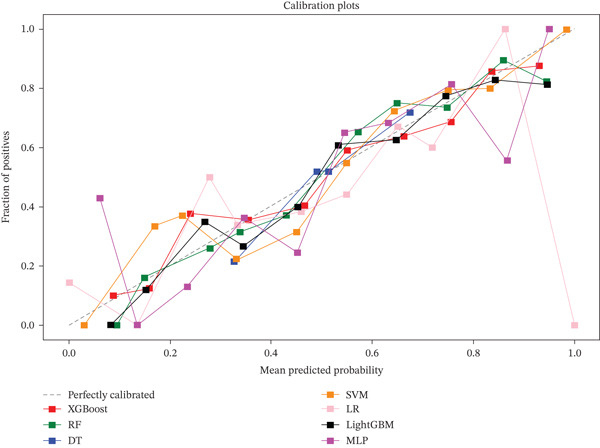
(b)

**Table 2:** Comparative analysis of performance results for different machine learning models.

Models	AUC	AUPRC	Accuracy	Sensitivity	Specificity	PPV	NPV	F1 Score	Brier score
RF	0.85	0.696	0.79	0.79	0.78	0.78	0.80	0.78	0.159
DT	0.68	0.430	0.68	0.72	0.63	0.65	0.71	0.69	0.301
XGBoost	0.84	0.678	0.75	0.77	0.73	0.73	0.77	0.75	0.179
SVM	0.75	0.582	0.70	0.75	0.64	0.67	0.73	0.71	0.203
LR	0.67	0.503	0.61	0.63	0.59	0.67	0.63	0.61	0.229
LightGBM	0.83	0.686	0.75	0.76	0.74	0.73	0.76	0.75	0.177
MLP	0.80	0.539	0.72	0.71	0.72	0.71	0.72	0.71	0.186

Abbreviations: AUC, the area under the receiver‐operating characteristic; AUPRC, the area under the precision‐recall curve; DT, decision tree; LightGBM, light gradient boosting machine; LR, logistic regression; MLP, multilayer perceptron; NPV, negative predictive value; PPV, positive predictive value; RF, random forest; SVM, support vector machine; XGBoost, eXtreme Gradient Boosting.

XGBoost (*A* *U* *C* = 0.84) and LightGBM (*A* *U* *C* = 0.83) showed comparable but consistently inferior performance to RF (*A* *U* *C* = 0.85) in terms of accuracy, sensitivity, and calibration (Brier score). SVM and MLP achieved moderate predictive performance, with AUCs of 0.75 and 0.80, respectively. Taken together, based on a comprehensive evaluation of AUC, prediction accuracy, and calibration reliability, RF was selected as the core model for subsequent interpretability analysis due to its optimal discriminative power and robustness.

To assess the clinical usefulness of the model, DCA was performed. The DCA showed that the RF model provided a higher net benefit than the “treat‐all” and “treat‐none” strategies across most clinically relevant threshold probabilities, particularly above approximately 0.05 (Figure S1). At very low threshold probabilities (< 0.05), the net benefit of the RF model was comparable with that of the treat‐all strategy.

### 3.4. External Validation of the Model

To assess the generalizability of the final RF model, an independent external validation was performed using data from 495 AdHF patients at Shanghai Tenth People′s Hospital. Table S2 compares baseline characteristics of the derivation and external validation cohorts, including the eight variables selected for model development. No statistically significant differences were observed for any of the included variables (all *p* > 0.05), suggesting good comparability between the two cohorts.

In the external validation cohort, the RF model demonstrated robust generalizability with an AUC of 0.76 (95% CI: 0.70–0.83; Figure S2). As shown in Table S3, RF also achieved the highest accuracy (0.69), specificity (0.72), and lowest Brier score (0.199) among all seven models, confirming its superior overall performance. RF also demonstrated the highest AUPRC (0.611), further confirming its robustness in identifying readmission risk (Table S3). Calibration plots (Figure S3) further indicate that RF maintained good probability calibration in the external cohort, with predicted probabilities closely aligning with observed outcomes. These findings reinforce that the RF model′s superiority is not limited to the derivation cohort but extends to an independent external population.

### 3.5. Global Interpretability of the Optimal Model

SHAP analysis was employed to globally interpret the optimal RF model, aiming to quantify the contribution of each feature to the prediction of 30‐day readmission risk in patients with AdHF. Figure 4a illustrates the mean absolute SHAP values, indicating the average impact of each variable on model output. Figure 4b presents the distribution of SHAP values, with dot colors reflecting feature magnitudes (red for high, blue for low) and their positions indicating the direction and magnitude of influence on the predicted outcome.

Figure 4Global model explanation by the SHAP method. (a) SHAP summary bar plot. (b) SHAP summary dot plot. Abbreviations: CRP, C‐reactive protein; TYG‐BMI, triglyceride‐glucose‐body mass index; NYHA, New York Heart Association; NLR, the neutrophil‐to‐lymphocyte ratio; AF, atrial fibrillation; ACEI, angiotensin‐converting enzyme inhibitor; ARB, angiotensin receptor blockers; ARNI, angiotensin receptor–neprilysin inhibitor.

**Figure:**
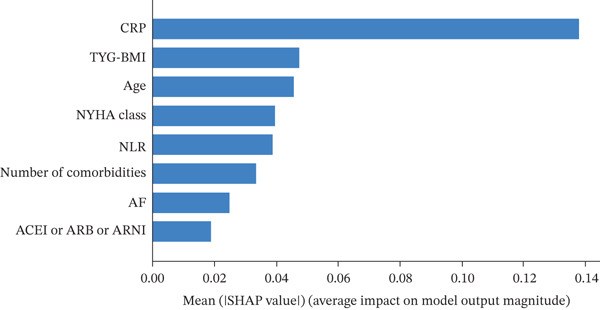
(a)

**Figure:**
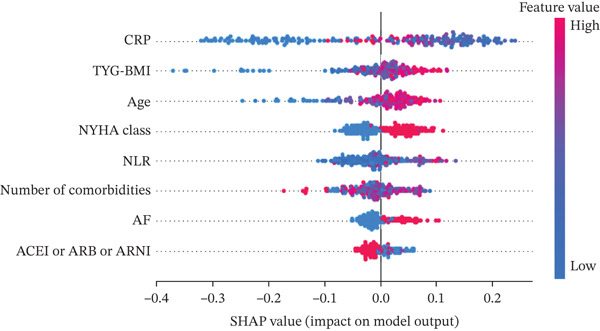
(b)

CRP and TyG‐BMI emerged as the most influential predictors; higher values were associated with greater positive SHAP values, suggesting a strong positive contribution to readmission risk. It is worth noting that while individual low values for CRP or Number of comorbidities may occasionally exhibit positive SHAP values due to complex interactions with other high‐risk features (e.g., advanced age or NYHA Class IV), the overall trend in Figure 4b demonstrates a clear positive association: increasing levels of these variables are consistently linked to elevated SHAP contributions to readmission risk. These findings underscore the pivotal role of inflammation and metabolic disturbance in AdHF‐related readmission. Other important variables, including age, NYHA, NLR, number of comorbidities, and AF, also showed positive correlations with readmission risk.

Notably, “ACEI or ARB or ARNI” was associated with predominantly negative SHAP values at higher feature levels, implying a potential protective effect against readmission. Overall, the SHAP plots provide a comprehensive understanding of how inflammation markers, metabolic burden, clinical status, comorbidities, and therapeutic interventions collectively influence the model′s prediction, offering valuable insights into the underlying mechanisms.

### 3.6. Clinical Utility of the Dynamic Nomogram

A dynamic nomogram (Figure 5) was developed by integrating eight key predictors, including CRP, TyG‐BMI, and age, to provide real‐time, individualized risk assessment for 30‐day readmission in AdHF patients. This interactive tool allows users to adjust input values via sliders or text boxes, with the system automatically calculating the corresponding risk probability. The online demonstration is available at: https://scinomogram.shinyapps.io/CTANNNAA/, offering an intuitive visualization of how parameter changes influence predicted outcomes.

Figure 5Dynamic nomogram for predicting a 30‐day readmission risk in AdHF patients. (a) Input page: enter the patient′s information according to the relevant variables on this page. (b) Graphical summary: this page shows the probability of a patient being readmitted to hospital with heart failure and the 95% confidence interval. (c) Numerical summary: display the specific values of the patient′s indicators and predicted outcomes.Abbreviations: NYHA, New York Heart Association; AF, atrial fibrillation; NLR, the neutrophil‐to‐lymphocyte ratio; TYG‐BMI, triglyceride‐glucose‐body mass index; CRP, C‐reactive protein; ACEI, angiotensin‐converting enzyme inhibitor; ARB, angiotensin receptor blockers; ARNI, angiotensin receptor–neprilysin inhibitor.

**Figure:**
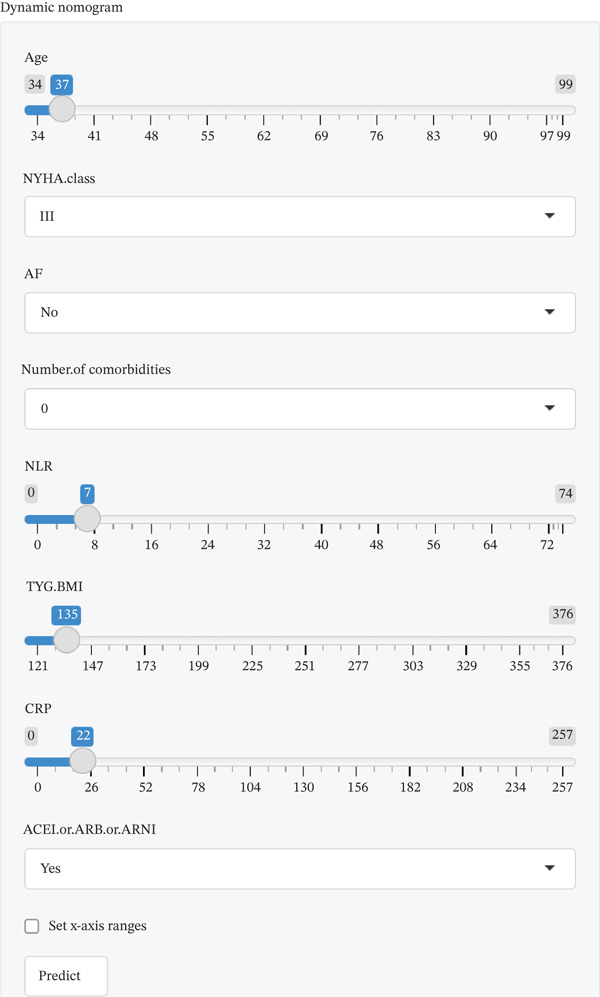
(a)

**Figure:**
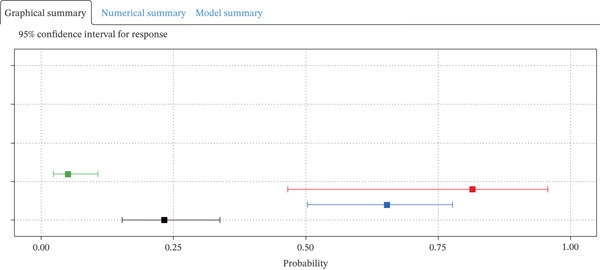
(b)

**Figure:**
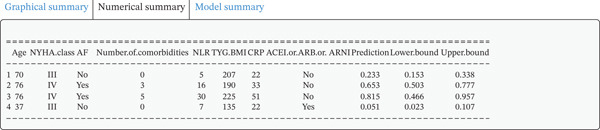
(c)

For instance, consider a 76‐year‐old patient with AdHF who presents with CRP of 51 mg/L, TyG‐BMI of 225, NYHA Class IV, NLR of 30, five comorbidities, AF, and no use of ACEI/ARB/ARNI. After inputting these values, the tool estimates a 30‐day readmission risk of 81.50%. This model can assist clinicians in tailoring interventions, such as optimizing anti‐inflammatory strategies or adjusting medications for high‐risk individuals, thereby supporting personalized management.

### 3.7. Supporting Results

To provide additional methodological transparency and support the robustness of the findings, several supporting tables are included. Table S1 details the full set of hyperparameters used in the final RF model. Table S2 presents a comparison of baseline characteristics between the derivation cohort and the external validation cohort, demonstrating good comparability across key clinical and biomarker variables. Table S3 summarizes the predictive performance of all machine learning models in the external validation cohort, further confirming the superior and stable performance of the RF model.

## 4. Discussion

Readmission not only imposes a significant burden on healthcare systems but is also closely associated with poor long‐term outcomes. In patients with AdHF, the complexity of disease management is further heightened by multisystem dysfunction and the fluctuating nature of their clinical course. This study focused on a 30‐day readmission among patients with AdHF after discharge, revealing a readmission rate of 30.9% (238/769), which is slightly higher than previously reported rates for HF patients (20.9%–23.6%) [14–16]. These findings underscore the urgent need for enhanced discharge planning and individualized interventions in this high‐risk population. By leveraging multiple ML algorithms, this study identified RF as the most robust model in terms of discrimination, calibration, and generalizability. The superior performance of RF enhances the precision of risk stratification and provides valuable insights to support early intervention and optimal resource allocation for high‐risk patients.

Among the numerous predictors, inflammatory and metabolic biomarkers demonstrated particularly strong predictive value in this study. CRP, a classical acute‐phase reactant, not only reflects systemic inflammatory activation but is also closely associated with myocardial injury, ventricular remodeling, and immune dysregulation [17, 18]. In this study, CRP exhibited the highest contribution in the SHAP analysis, reinforcing its role as a key biomarker for short‐term adverse outcomes. NLR, another critical inflammatory indicator, integrates information on both inflammation and immune response. Previous studies have demonstrated that elevated NLR levels are closely associated with an increased risk of readmission in HF patients and are significantly correlated with a 1‐year all‐cause mortality in AdHF patients [19, 20]. Our findings revealed a positive association between elevated NLR and 30‐day readmission risk, suggesting that immune‐inflammatory imbalance may play a facilitating role in the pathophysiology of AdHF, particularly in the context of multimorbidity, where its clinical sensitivity is amplified. On the metabolic side, TyG‐BMI, a composite marker of IR and lipotoxic burden, also emerged as a significant contributor in the predictive model [13]. Prior studies have demonstrated its utility in cardiovascular risk stratification, and our results further confirmed its nonlinear association with readmission risk, supporting its potential as a valuable tool for monitoring metabolic stress [21].

In addition to inflammatory and metabolic markers, age and NYHA class, both key indicators of baseline condition and HF severity, demonstrated stable predictive value in this study. Elderly patients often exhibit impaired immune function, and coexistence of multiple chronic diseases, all of which substantially increase the risk of readmission [22, 23]. We observed that higher age and more advanced NYHA class were significantly associated with increased readmission risk, consistent with previous findings [24]. Notably, LVEF did not emerge as a predictor in our model, likely reflecting its limited discriminatory power in an AdHF population where most patients already exhibit severely reduced systolic function. Moreover, AF and the number of comorbidities, as indicators of multimorbidity, were also strongly associated with short‐term readmission. As a common arrhythmia in HF patients, AF not only exacerbates cardiac dysfunction but may also increase readmission risk due to bleeding complications related to anticoagulation therapy and poor treatment adherence [25–27]. Our study further revealed that patients with two or more comorbidities had a significantly elevated risk of readmission, highlighting the substantial burden that multimorbidity imposes on clinical management. With the ongoing trend of population aging, multimorbidity has become increasingly prevalent in HF patients and holds growing significance in risk prediction models. Finally, the use of ACEI, ARB, or ARNI was associated with a lower risk of readmission, which is consistent with previous studies [28, 29]. ACEI/ARB/ARNI help slow HF progression by reducing neurohormonal activation, lowering cardiac afterload, and improving left ventricular remodeling [30, 31]. SHAP analysis also indicated a potential protective effect of these medications.

Although this study focused on 30‐day readmission as a clinically meaningful short‐term outcome, the proposed modeling framework may be extendable to longer‐term outcomes, such as a 90‐day or 1‐year readmission or mortality. However, extending prediction to longer horizons introduces additional challenges, including increased heterogeneity in post‐discharge management, changes in medical therapy over time, and the influence of competing risks such as death. Moreover, long‐term outcomes require more complete follow‐up data and careful handling of time‐dependent covariates to avoid bias. Future studies with longitudinal data and dynamic modeling approaches may help address these challenges and further enhance the clinical applicability of the proposed model.

## 5. Limitations

This study has several limitations. First, this study is retrospective, which is prone to selection bias and unmeasured confounding. However, given the low incidence of AdHF and the need for sufficient 30‐day readmission events to develop a reliable prediction model, a retrospective design spanning 2015–2024 was necessary. Second, the extended study period may have introduced temporal heterogeneity in clinical practice, as HF therapies such as ARNI and SGLT2 inhibitors became increasingly adopted over time. To minimize potential bias related to evolving therapeutic strategies during the long study period, we applied strict internal validation procedures, including tenfold cross‐validation for LASSO feature selection, application of SMOTE oversampling within the training set only, and model performance evaluation on a hold out validation set. Furthermore, external validation using an independent dataset from Shanghai Tenth People′s Hospital supported the model′s robustness and generalizability across different clinical environments. Given that the major predictors in our model are biologically based inflammatory and metabolic biomarkers, which are less affected by treatment evolution, the risk of performance inflation due to therapy advancement was considered limited. Third, only inflammatory and metabolic biomarkers measured at hospital admission were included. Although serial measurements (e.g., posttreatment or discharge values) and their temporal trajectories may offer incremental prognostic value, our primary aim was to develop an admission‐based model to enable early risk stratification and timely clinical decision‐making. Future prospective studies integrating serial biomarker measurements and trajectory‐based modeling are warranted to further enhance predictive accuracy and clinical applicability. Fourth, although SMOTE was applied only to the training set, it may have influenced the joint distribution of features, potentially contributing to minor calibration deviations. Future work could explore alternative imbalance‐handling techniques (e.g., ensemble‐based resampling or cost‐sensitive learning) that better preserve data structure. Fifth, although NT‐proBNP and high‐sensitivity troponin are well‐established prognostic biomarkers in HF, our institution routinely measured BNP rather than N‐terminal pro‐B‐type natriuretic peptide (NT‐proBNP) during the study period. Troponin assays were not uniformly performed across all patients, resulting in a high rate of missing data (> 30%), which precluded their inclusion in the analysis.

## 6. Conclusion

This study developed a 30‐day readmission risk prediction model for AdHF patients, with RF showing the best performance. Inflammatory and metabolic markers (CRP, TyG‐BMI, and NLR), along with clinical variables like age, NYHA class, AF, the number of comorbidities, and medication use, were key predictors. SHAP analysis enhanced model interpretability, and a dynamic nomogram enables real‐time risk estimation. The model provides a practical tool for individualized risk assessment and targeted intervention in AdHF patients.

## Author Contributions

All authors made a significant contribution to the work reported, whether that is in the conception, study design, execution, acquisition of data, analysis and interpretation, or in all of these areas; took part in drafting, revising or critically reviewing the article.

## Funding

This study was supported by the Science and Technology Project of the Xuzhou Health Commission (No. XWKYHT20230074).

## Disclosure

All authors gave final approval of the version to be published; have agreed on the journal to which the article has been submitted; and agree to be accountable for all aspects of the work.

## Ethics Statement

The study protocol complied with the Declaration of Helsinki and received ethical approval from the institutional review boards of both the Affiliated Hospital of Xuzhou Medical University (XYFY2025‐KL089‐01) and Shanghai Tenth People′s Hospital (SHSY‐LYZX‐220). The requirement for informed consent was waived by the ethics committee due to the retrospective nature of the study and the anonymization of patient data. All patient data were strictly kept confidential and used solely for this research. The study adhered to relevant ethical standards, ensuring the privacy and security of patient information.

## Conflicts of Interest

The authors declare no conflicts of interest.

## Supporting Information

Additional supporting information can be found online in the Supporting Information section.

## Supporting information


**Supporting Information 1** Figure S1: DCA for the RF model. The net benefit (*y*‐axis) is plotted against the threshold probability (*x*‐axis), which represents the minimum risk at which a clinician would recommend intervention. Three strategies are compared: (1) Random forest (blue): uses the model to guide decisions; (2) treat all (red): treats every patient; and (3) treat none (black): treats no one.

**Supporting Information 2** Figure S2: ROC curve for the RF model in the external validation cohort. Abbreviation: AUC, area under the receiver operating characteristic.

**Supporting Information 3** Figure S3: Calibration curves for the RF model in the external validation cohort. Abbreviations: AUC, the area under the receiver‐operating characteristic; CI, confidence interval; RF, random forest; DT, decision tree; XGBoost, eXtreme Gradient Boosting; SVM, support vector machine; LR, logistic regression; LightGBM, light gradient boosting machine; MLP, multilayer perceptron.

**Supporting Information 4** Table S1: Hyperparameters of the final random forest model.

**Supporting Information 5** Table S2: Baseline characteristics of patients in the derivation cohort and the external validation cohort.

**Supporting Information 6** Table S3: Comparative analysis of performance results for different machine learning models in the external.

## Data Availability

The raw data supporting the conclusions of this article will be made available by the authors without undue reservation.
