# Supplementary figures and images for: Interpretable Machine Learning Model for Predicting 30‐Day Readmission in Advanced Heart Failure Patients: Synergistic Assessment of Inflammatory and Metabolic Biomarkers
Source: Cardiovasc Ther. 2026 Mar 8;2026:2307901. doi: 10.1155/cdr/2307901 (PMC12968333; doi:10.1155/cdr/2307901)

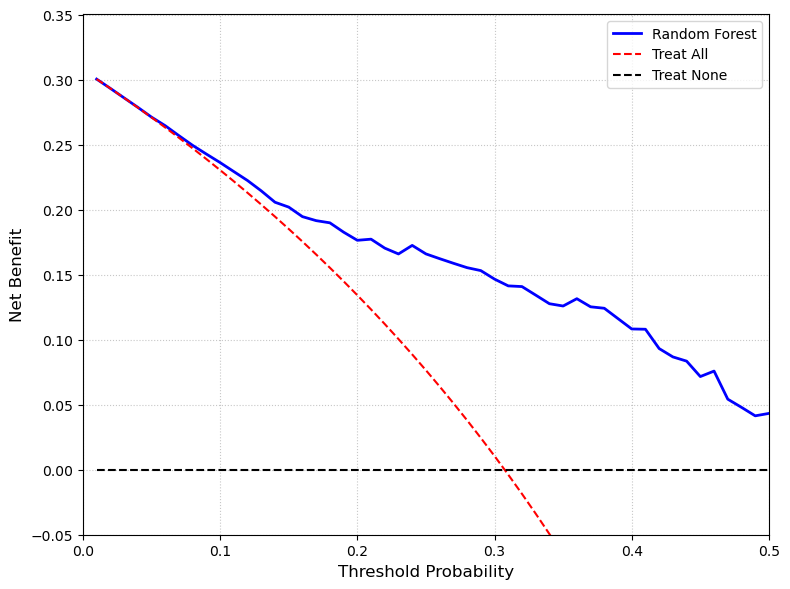

Supplement: Supplementary file 1 — Supporting Information 1 Figure S1: DCA for the RF model. The net benefit (y‐axis) is plotted against the threshold probability (x‐axis), which represents the minimum risk at which a clinician would recommend intervention. Three strategies are compared: (1) Random forest (blue): uses the model to guide decisions; (2) treat all (red): treats every patient; and (3) treat none (black): treats no one. [file CDR-2026-2307901-s005.png]

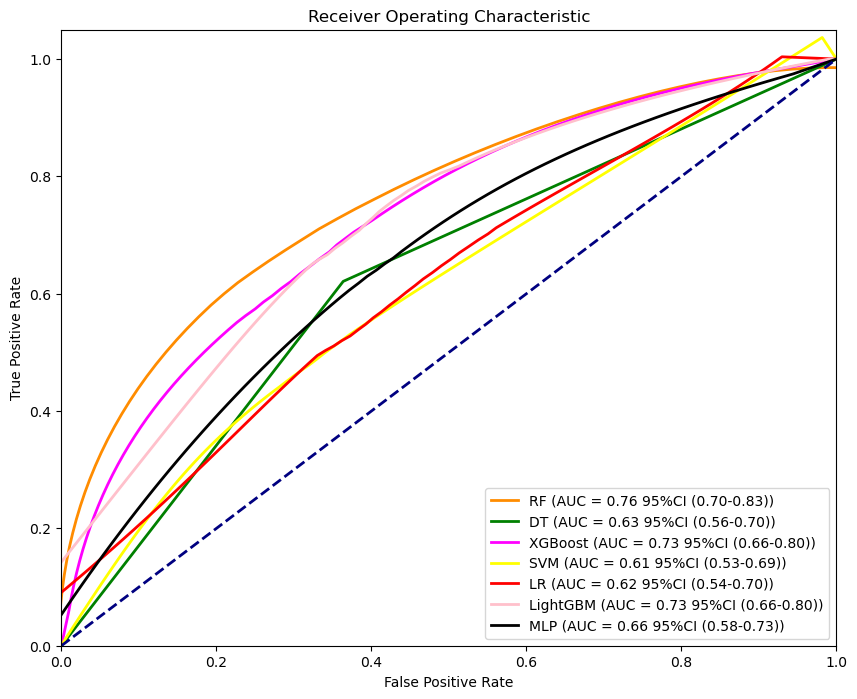

Supplement: Supplementary file 2 — Supporting Information 2 Figure S2: ROC curve for the RF model in the external validation cohort. Abbreviation: AUC, area under the receiver operating characteristic. [file CDR-2026-2307901-s003.png]

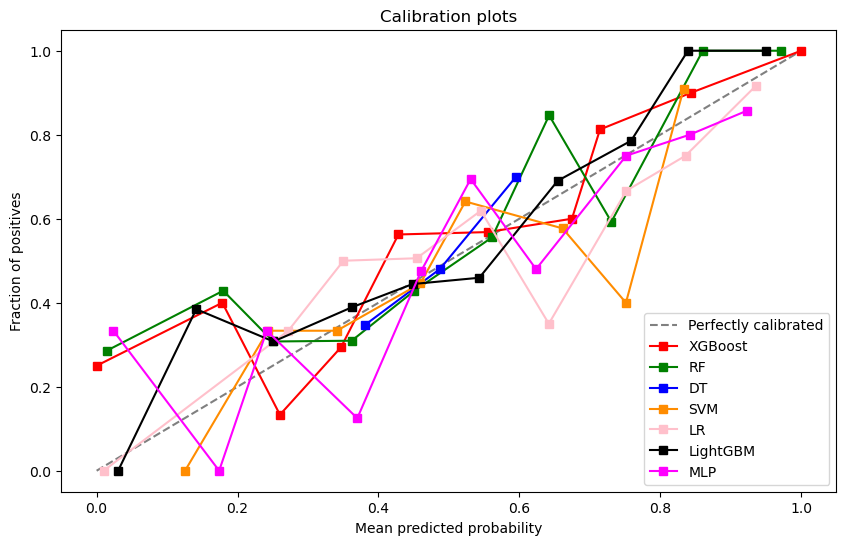

Supplement: Supplementary file 3 — Supporting Information 3 Figure S3: Calibration curves for the RF model in the external validation cohort. Abbreviations: AUC, the area under the receiver‐operating characteristic; CI, confidence interval; RF, random forest; DT, decision tree; XGBoost, eXtreme Gradient Boosting; SVM, support vector machine; LR, logistic regression; LightGBM, light gradient boosting machine; MLP, multilayer perceptron. [file CDR-2026-2307901-s001.png]
