# Supplementary material for: Interpretable Machine Learning Model for Predicting 30‐Day Readmission in Advanced Heart Failure Patients: Synergistic Assessment of Inflammatory and Metabolic Biomarkers
Source: Cardiovasc Ther. 2026 Mar 8;2026:2307901. doi: 10.1155/cdr/2307901 (PMC12968333; doi:10.1155/cdr/2307901)
Supplement: Supplementary file 4 — Supporting Information 4 Table S1: Hyperparameters of the final random forest model. [file CDR-2026-2307901-s002.docx]

| Parameter | Value | Description |
| --- | --- | --- |
| n_estimators | 100 | Number of decision trees in the forest |
| criterion | gini | Function to measure the quality of a split |
| max_depth | None | Maximum depth of the tree (no limit imposed) |
| min_samples_split | 2 | Minimum number of samples required to split an internal node |
| min_samples_leaf | 1 | Minimum number of samples required to be at a leaf node |
| max_features | sqrt | Number of features considered for best split |
| bootstrap | True | Whether bootstrap samples are used when building trees |
| class_weight | None | Class weights not applied (class imbalance handled via SMOTE) |
| random_state | 42 | Seed for reproducibility |
| oob_score | False | Out-of-bag score not computed |
| ccp_alpha | 0.0 | Complexity parameter used for Minimal Cost-Complexity Pruning (none applied) |
| max_leaf_nodes | None | No constraint on the maximum number of leaf nodes per tree |
| min_impurity_decrease | 0.0 | Split only if impurity decrease ≥ this value |
| max_samples | None | All samples used for bootstrap (no subsampling) |

Supplementary Table 1. Hyperparameters of the Final Random Forest Model.
