# Supplementary material for: Interpretable Machine Learning Model for Predicting 30‐Day Readmission in Advanced Heart Failure Patients: Synergistic Assessment of Inflammatory and Metabolic Biomarkers
Source: Cardiovasc Ther. 2026 Mar 8;2026:2307901. doi: 10.1155/cdr/2307901 (PMC12968333; doi:10.1155/cdr/2307901)
Supplement: Supplementary file 5 — Supporting Information 5 Table S2: Baseline characteristics of patients in the derivation cohort and the external validation cohort. [file CDR-2026-2307901-s006.docx]

| Supplementary Table 2. Baseline Characteristics of Patients in the Derivation Cohort and the External Validation Cohort. | | | |
| --- | --- | --- | --- |
| Variables | Derivation Cohort  (n=769) | External Validation Cohort  (n=495) | *P-*Value |
| Age | 70.35±11.89 | 71.43±11.47 | 0.114 |
| NYHA class (n, %) |  |  | 0.544 |
| III | 431 (56.0%) | 286 (57.8%) |  |
| IV | 338 (44.0%) | 209 (42.2%) |  |
| Past medical history (n, %) |  |  |  |
| AF | 222 (28.9%) | 160 (32.3%) | 0.192 |
| Numberofcomorbidities |  |  | 0.964 |
| 0 | 135 (17.6%) | 80 (16.2%) |  |
| 1 | 280 (36.4%) | 178 (36.0%) |  |
| 2 | 190 (24.7%) | 130 (26.3%) |  |
| 3 | 116 (15.1%) | 79 (16.0%) |  |
| 4 | 40 (5.2%) | 23 (4.6%) |  |
| 5 | 8 (1.0%) | 5 (1.0%) |  |
| Indices |  |  |  |
| NLR | 3.46 (2.30,5.33) | 3.37 (2.42,4.74) | 0.450 |
| TYG-BMI | 202.50 (179.16,231.50) | 202.22 (179.59,229.67) | 0.870 |
| CRP (mg/L) | 8.39 (2.11,24.19) | 8.45 (2.18,23.25) | 0.927 |
| Inotropic drugs (n, %) |  |  |  |
| ACEI/ARB/ARNI | 520 (67.6%) | 342 (69.1%) | 0.584 |

Abbreviations: NYHA, New York Heart Association; AF, atrial fibrillation; NLR, the neutrophil-to-lymphocyte ratio; TYG-BMI, triglyceride-glucose-body mass index; CRP, C-reactive protein; ACEI, angiotensin-converting enzyme inhibitor; ARB, angiotensin receptor blockers; ARNI, angiotensin receptor-neprilysin inhibitor.
