# Supplementary material for: Interpretable Machine Learning Model for Predicting 30‐Day Readmission in Advanced Heart Failure Patients: Synergistic Assessment of Inflammatory and Metabolic Biomarkers
Source: Cardiovasc Ther. 2026 Mar 8;2026:2307901. doi: 10.1155/cdr/2307901 (PMC12968333; doi:10.1155/cdr/2307901)
Supplement: Supplementary file 6 — Supporting Information 6 Table S3: Comparative analysis of performance results for different machine learning models in the external. [file CDR-2026-2307901-s004.docx]

Supplementary Table 3. Comparative Analysis of Performance Results for Different Machine Learning Models in the external validation cohort.

| Models | AUC | AUPRC | Accuracy | Sensitivity | Specificity | PPV | NPV | F1 Score | Brier Score |
| --- | --- | --- | --- | --- | --- | --- | --- | --- | --- |
| RF | 0.76 | 0.611 | 0.69 | 0.67 | 0.72 | 0.72 | 0.67 | 0.70 | 0.199 |
| DT | 0.63 | 0.407 | 0.63 | 0.62 | 0.64 | 0.65 | 0.61 | 0.63 | 0.372 |
| XGBoost | 0.73 | 0.564 | 0.66 | 0.67 | 0.65 | 0.67 | 0.65 | 0.67 | 0.252 |
| SVM | 0.61 | 0.541 | 0.56 | 0.57 | 0.56 | 0.58 | 0.54 | 0.57 | 0.239 |
| LR | 0.62 | 0.557 | 0.6 | 0.61 | 0.59 | 0.58 | 0.58 | 0.61 | 0.237 |
| LightGBM | 0.73 | 0.529 | 0.65 | 0.68 | 0.61 | 0.66 | 0.64 | 0.67 | 0.229 |
| MLP | 0.66 | 0.478 | 0.63 | 0.67 | 0.58 | 0.63 | 0.62 | 0.65 | 0.246 |

Abbreviations: RF, Random Forest; DT, Decision Tree; XGBoost, extreme gradient Boosting; SVM, Support Vector Machine; LR, Logistic Regression; LightGBM, light gradient boosting machine; MLP, Multilayer Perceptron; AUC, the area under the receiver-operating characteristic; AUPRC, the area under the precision-recall curve; PPV, positive predictive value; NPV, negative predictive value.
